# Sutureless technique to support anastomosis during thoracic aorta replacement

**DOI:** 10.1186/1749-8090-4-66

**Published:** 2009-11-13

**Authors:** Efstratios E Apostolakis, Vassilios N Leivaditis, Constantinos Anagnostopoulos

**Affiliations:** 1 Department of Cardiothoracic Surgery, Patras University School of Medicine, Patras, Greece

## Abstract

**Background:**

In aortic replacement procedures the aortic wall and Teflon strips form a double layer, with the use of continuous sutures. Surgical glues may or may not be used to enhance the durability of the anastomoses. In this technical report a modification of the aortic stumps preparation is devised.

The technique reduces substantially the preparation time of the aortic stumps by the use of ligation clips and a surgical sealant.

**Technique:**

Suturing is the standard method for the aortic-teflon double-layer formation prior to Dacron anastomosis. In this study, instead of suturing, 5-6 ligation clips are primarily applied on the exterior of the double layer to facilitate proper cooptation. Secondarily, in order to fuse the two layers together, a sealant is injected in between the Teflon and aortic wall. Thus each stump is delivered quickly sutureless for the Dacron anastomosis.

Between January 2003 and March 2009 this modified operative technique was performed in 14 cases (group A) with a mean age of 50 ± 16 years. This was contrasted against 24 controls (group B), with a mean age of 40 ± 28 years, treated with the conventional method, where only continuous sutures were used during the anastomosis. All patients were cases of ascending aorta replacement and/or aortic hemi-arch replacement, for acute aortic dissection or aortic dilatation.

**Results:**

The pure anastomosis time (stump preparation and Dacron connection) was shortened by approximately 25 minutes depending on surgeon's experience. The anastomosis blood-loss was also significantly reduced in the sutureless group A, as evident by the dry operative field and the limited use of blood products, post-prosthetic graft anastomosis. This reflected to a faster post-operative recovery, faster extubation and fewer complications. At a mean follow-up of 21 ± 7 days, there were no post-operative deaths being related to acute aortic dissection or rupture of the anastomotic site.

**Conclusion:**

Aortic replacement with the combination of ligation clips and a surgical sealant vs. sutures alone allows easy manipulations of the aorta and adaptation of the diameters, thus optimizing aortic operational timings and hemostasis. Moreover, it prevents blood loss and aortic wall trauma from multiple sutures.

## Background

All types of thoracic aorta replacement carry a significant risk of post-operative bleeding resulting in either death or prolonged hospitalization, with an incidence as high as 24% [1]. Although, in a minority of patients, the aortic wall can be remarkably normal, most frequently the dissected and friable aortic wall extends into the anastomotic area, making the reconstruction and perfect sealing of the aorta extremely difficult.

For that main reason the aortic stumps -dissected or not- are routinely tightened with Teflon strips and biologic glue [1-4]. Usually, either two strips of synthetic felt are sutured on the interior and exterior of the aortic margin (sandwich technique), or alternatively a sole strip is used into the dissection [2,3]. However in either operative technique, the sutures create new holes in the already traumatized aortic wall and subsequent bleeding may be unavoidable. In an attempt to minimize the aortic wall damage, we have recently developed a modified sutureless technique of aortic stumps preparation. The report describes our experience with it and explains why contributes to faster, easier and better sealed anastomosis.

## Technique

**Figure 1:**
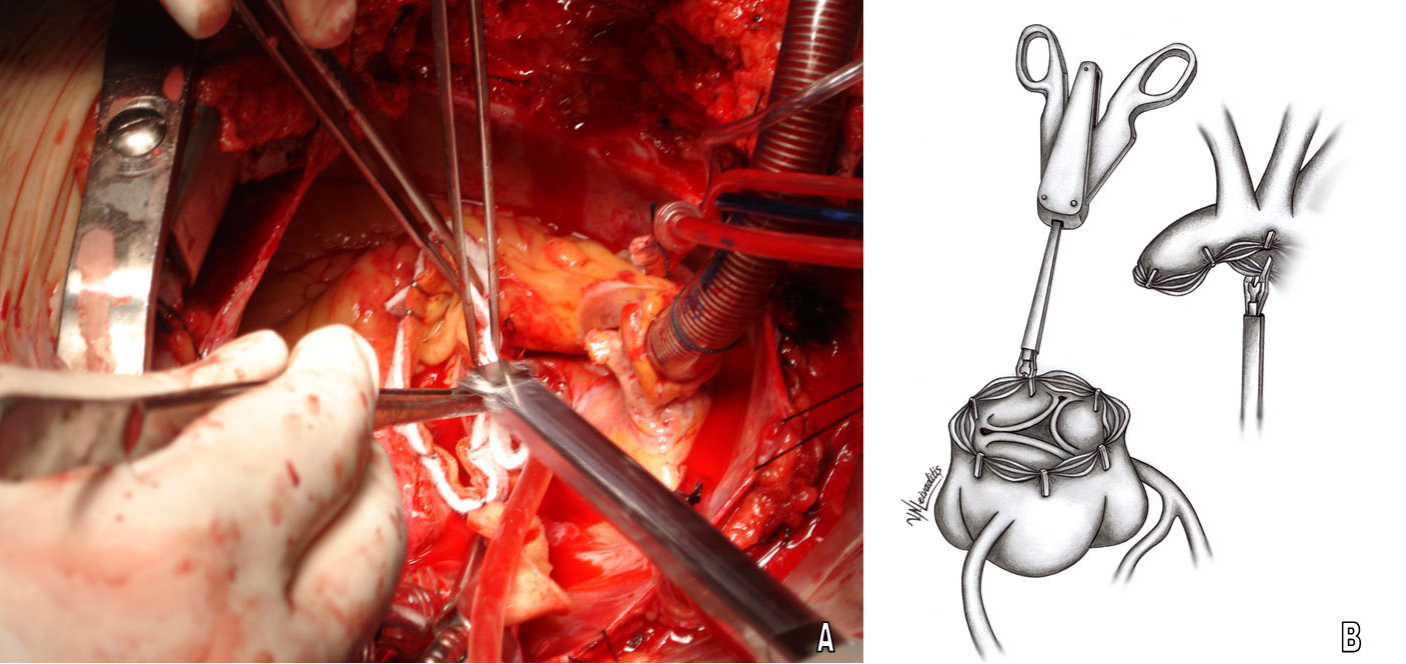
**Application of stapler for Teflon strips fixation**. A. Surgical field image, B. Graphic design representing the technique.

The thoracic aorta is transected just beyond the aneurysmal dilatation and the diseased part is removed. Two long strips of Teflon (0.8-1 cm width) are tailored to correspond to the circumference of the proximal and distal aortic stump. A 25 mm automatic stapler applies 9.8 mm large ligation clips (ETHICON^®^, LIGACLIP^®^, MCA, USA) at five to eight sites along the periphery of each stump (Figure 1). Care is taken to space the clips in such a fashion so as to avoid loops and gaps of the strips. The left ventricle (LV) is vented with a suction cannula inserted through the AV. A small piece of gauge with a central whole at the level of the AV prevents accidental fall of the clips in the LV. In the event of acute aortic dissection a single strip of Teflon between the two layers of the dissected aortic wall is used, applying the same principle.

**Figure 2:**
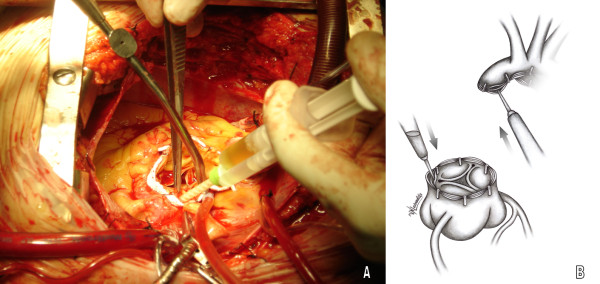
**Application of biologic glue between Teflon strips and aortic wall**. A. Surgical field image, B. Graphic design representing the technique.

**Figure 3:**
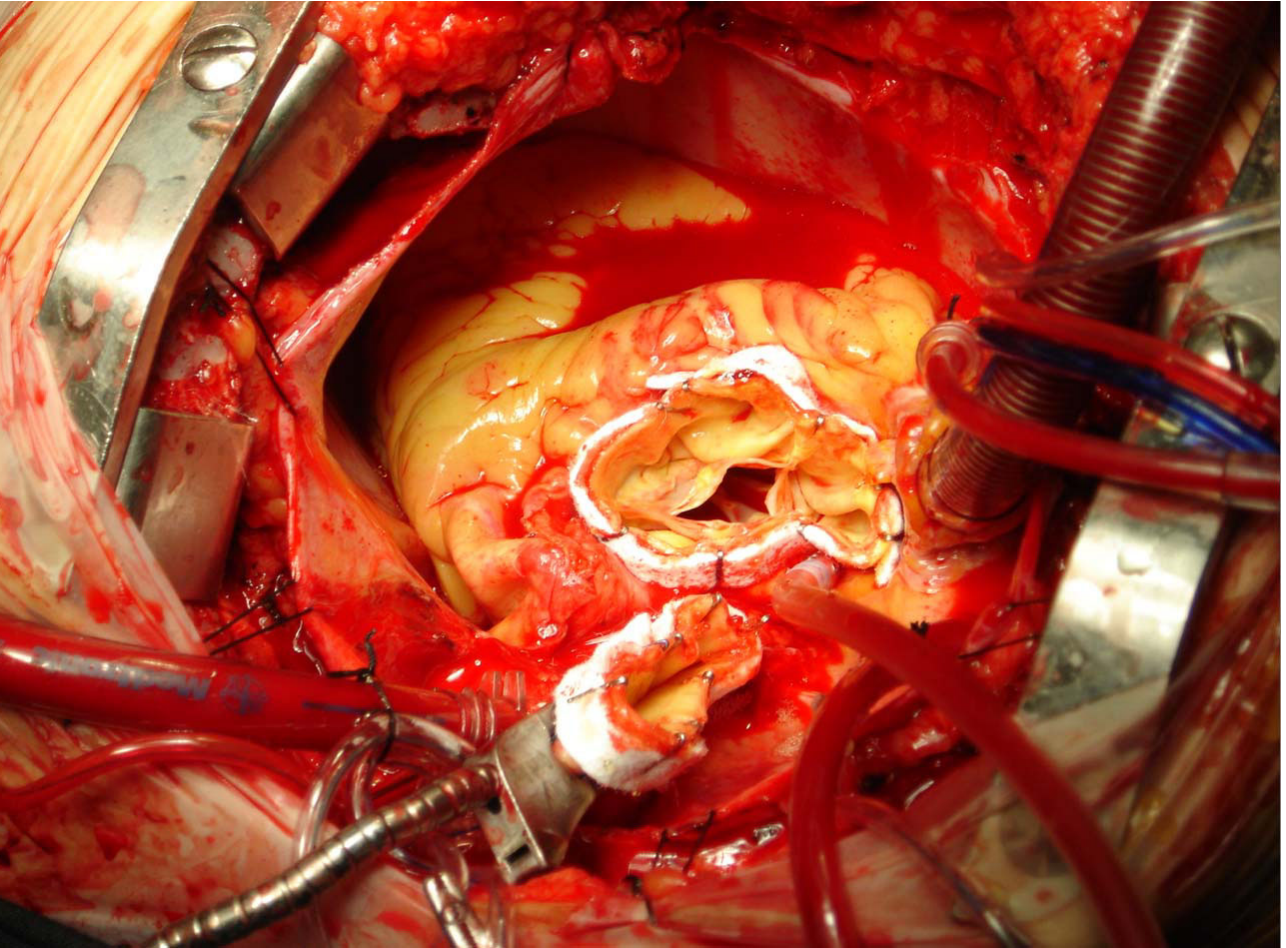
**The two stumps of the ascending aorta after application of the glue and before removing the clips**.

**Figure 4:**
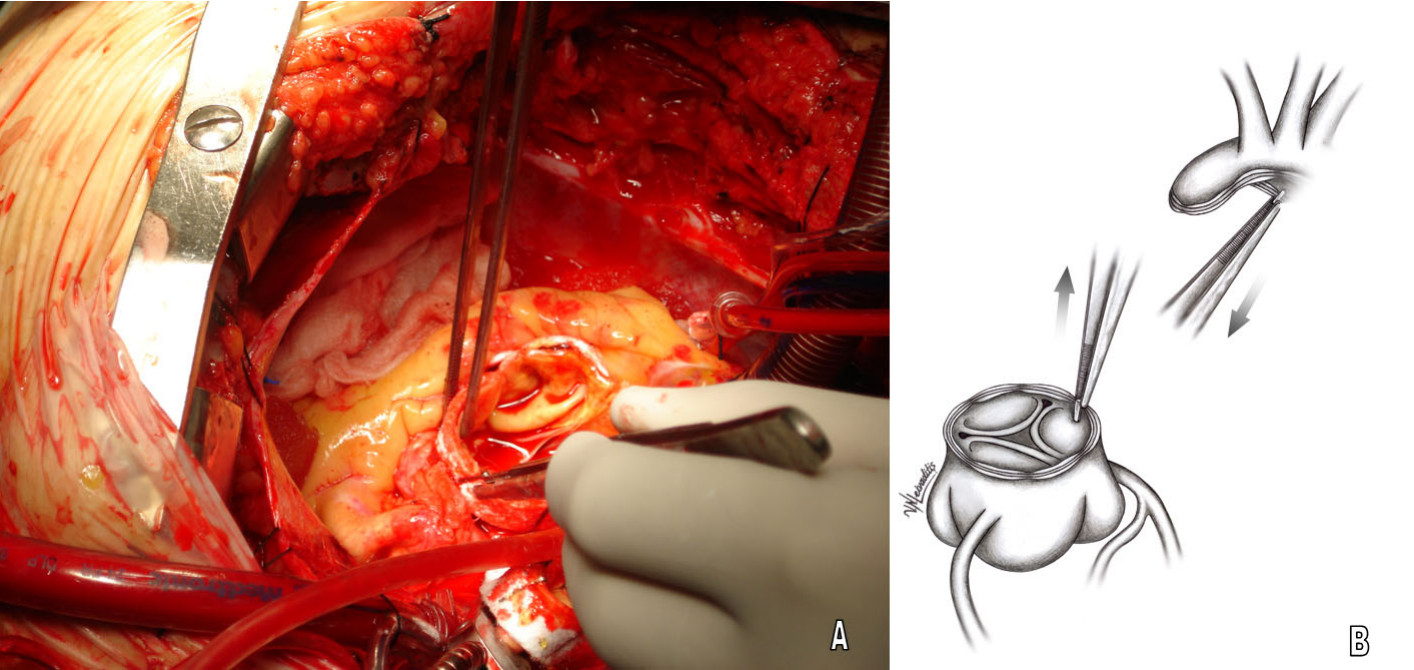
**Removing out of ligation clips from the aortic stumps**. A. Surgical field image, B. Graphic design representing the technique.

In as much as the teflons strip surface a thin layer of glue is injected and left to fuse (Gelatin resorcinol formalin glue GRF^®^, Cardial, Technopole, France or BioGlue BIOGLUE^® ^Surgical Adhesive glue, CryoLife, USA) (figure 2, 3). The edges of the remodelled aortic portions are subsequently sewn to the Dacron graft -at the appropriate level- with a continuous 4-0 Prolene suture. The clips are easily removed with a gentle pull during the anastomosis (figure 4).

It is a matter of judgement as to how much glue needs to be injected. In some cases the extended use of biologic glue is regarded as precarious. Although no complication has been reported as yet, reservations relating to the use of glue still remain.

The above technique allows exact cooptation and facilitates suturing, regardless the use of glue. Stay clips may always be used to optimize symmetric positioning and proper Teflon structural adaptation; they can be easily removed in the course of the Dacron- Aortic anastomosis.

## Results

This is a limited technical report with non-randomized character of 14 cases and 24 controls.

The small number of cases combined with secondary factors (such as: variability of arch or hemiarch involvement, interpositional or composite graft usage, complications of transfemoral bypass, perioperative coagulopathy, pre-op anticoagulation treatment, emergency character of dissecting aneurysms etc) prevented a statistically safe evaluation at this stage. Although detailed parametric analysis (cross clamp time, CPB-time, circulatory arrest-time, perioperative blood losses) and mid-term dissecting complications rate are eagerly expected from the completion of a new randomized study, our preliminary data reveal quantifiable trends towards a significant suturing time reduction, easier anastomosis and improved intraoperative hemostasis.

We propose that this new method can be safely applied on the base of absence of complications and improved operative feasibility.

## Discussion

The reinforcement of suture-line by using Teflon strips with or without biologic glue is a method of wide acceptance in aortic replacement which aims to faster operation and better sealing [4-6]. Fixation of Teflon strips on the stumps is achieved by using an "over and over" or a "meander-like" continuous suture [1-3]. This is a demanding surgical manoeuvre extending the CPB time by a factor of 15 to 30 minutes contributing significantly to the frailness of the aortic wall.

The friability of a diseased aortic wall is regarded as a dominant problem in the replacement of any part of the aorta. Although friability results mainly from underlying tunica media disease and substantial aneurysmal dilation, it is also affected by the repetitive suturing and confers significantly into perioperative bleeding. Especially when combined with deranged clotting, long CPB time and hypothermia form a true vicious circle with very adverse outcome.

The technique described above diminishes considerably the stump preparation-time, contributes to reduced friability and is expected to contribute to better short term survival.

The use of bulldogs instead of ligation clips has been described in surgical textbooks and could certainly be an alternative option. However their size makes the surgical manipulations much harder. In addition, their use demands that the assistant holds them in certain position until the glue sets.

According to Kunihara et al, the adjuvant to the biologic glue reinforcement using felt strips provides better midterm results [9]. Several studies have demonstrated that the non use of biologic glue during surgical reconstruction of the acute aortic dissection of the thoracic aorta is highly associated with rupture of the aortic false lumen [7,8]. However a few of our (and others') initial concerns, had been whether the unrestricted use of glue will convert to rigid the stump-Teflon double layer, or impede the suturing. Although this fear proved inaccurate, some surgeons may address concerns.

It is important to emphasize that applying the surgical clips -with or without the additive "protection" of glue- provides definite means of enhancing the aortic stump's support and results in a faster and easier anastomosis.

## Conclusion

Decreasing surgical time during replacement of any part of the thoracic aorta, in correlation with anastomosis tightness so as to avoid any possible leakage, is always a severe concern in related operations. The proposed method offers such valuable advantages to the patient. The use of temporary clips to fix the teflon strips, until the glue material sets, offers some important decrease to the time needed to perform the anastomoses, since the surgeon avoids the necessity to add the classical preparative sutures to the aortic stumps.

## Competing interests

The authors declare that they have no competing interests.

## Authors' contributions

EA conceived the idea, was the surgeon who performed the operations, wrote the first draft and led the project from beginning to end. VL assisted the study in data collection, literature review, draft revision, figure design and coordinating with all co-authors. CA helped with discussions about the topic offered useful suggestions and assisted in manuscript writing, reviewing the final draft. All authors critically read, discussed and approved the final draft of the manuscript.
